# On Pulse-Breath

**Published:** 1863-11

**Authors:** C. Radclyffe Hall

**Affiliations:** Physician to the Hospital for Consumption, Torquay


### ON PULSE-BREATH.

BY C. RADCLYFFE HALL, M.D., F.R.C.P., PHYSICIAN TO THE HOSPITAL FOR CONSUMPTION, TORQUAY.

Dr. Radclyffe Hall, by the term "pulse-breath," signifies an audible pulsation communicated to the breath as it issues from the mouth, by each beat of the heart. The sound is that of a gentle gushing of the breath, synchronous with each pulsation of the heart, and the degree of audibility varies in different cases and in the same case under varying circumstances. Dr. Hall has heard it so loud, that he could count the pulse by it at a distance of fifteen feet, and on the other hand it has been so subdued, that it was requisite to listen close to the patient's face for its detection. He heard it distinctly in two cases of phthisis in an advanced stage, and he considered that the mechanism of the production of the phonomenon is easily explained. A phthisical cavity old enough to possess rather dense walls and tolerably dry, by being emptied of its customary contents, and not immediately separated from the heart by permeable lung-tissues, vibrates with each beat of the heart, and at each vibration throws the air in the cavity, trachea, larynx, and mouth into a sonorous pulsation. When the cavity is more or less filled with liquid, it no longer vibrates, and as this is the habitual state of a cavity which has not collapsed, the phenomenon of "pulse-breath" is a rare occurrence. But Dr. Hall observed the same; phenomenon in a totally different case — namely, one of cardiac disease with enlarged liver, pulmonic congestion, and general anasarca. In the latter instance the explanation is more difficult than when a phthisical cavity exists, but the sound is supposed to be due to the impulse of the heart conveyed through the bloodvessels to the air-cells and passages. "Pulse-breath" has been hitherto undescribed by authors.—*Medico-Chirurgical Review.*

